# Psychometric validation of the Chinese version of the PaArticular Scales among elderly residents in long-term care facilities with joint contractures

**DOI:** 10.1186/s12877-021-02297-5

**Published:** 2021-06-09

**Authors:** Yi-chang Chen, Keh-chung Lin, Chen-Jung Chen, Shu-Hui Yeh, Ay-Woan Pan, Hao-Ling Chen, Chih-Hung Wang

**Affiliations:** 1 School of Occupational Therapy, College of Medicine, National Taiwan University, F4, No. 17, Xuzhou Rd., Zhongzheng Dist., Taipei, Taiwan; 2 Division of Occupational Therapy, Department of Physical Medicine and Rehabilitation, National Taiwan University Hospital, 46, Sec. 3, Zhongzheng Rd., Sanzhi Dist., Taipei, Taiwan; 3 Department of Nursing, Mackay Medical College, 46, Sec. 3, Zhongzheng Rd., Sanzhi Dist., Taipei, Taiwan; 4 Institute of Long-term Care, Mackay Medical College, Taipei, Taiwan; 5 Division of Occupational Therapy, Department of Physical Medicine and Rehabilitation, National Taiwan University, Taipei, Taiwan; 6 Graduate Institute of Education, National Changhua University of Education, No. 1, Jin-De Road, Changhua City, Taiwan

**Keywords:** Activity, Elderly residents, Joint contractures, Long-term care facilities, Participation, Reliability, Validity

## Abstract

**Background:**

Joint contractures, which affect activity, participation, and quality of life, are common complications of neurological conditions among elderly residents in long-term care facilities. This study examined the reliability and validity of the Chinese version of the PaArticular Scales in a population with joint contractures.

**Methods:**

A cross-sectional study design was used. The sample included elderly residents older than 64 years with joint contractures in an important joint who had lived at one of 12 long-term care facilities in Taiwan for more than 6 months (*N* = 243). The Chinese version of the PaArticular Scales for joint contractures was generated from the English version through five stages: translation, review, back-translation, review by a panel of specialists, and a pretest. Test-retest reliability, internal consistency reliability, construct validity, and criterion validity were evaluated, and the results were compared with those for the World Health Organization Quality of Life scale and the World Health Organization Disability Assessment Schedule.

**Results:**

The Chinese version of the PaArticular Scales had excellent reliability, with a Cronbach α coefficient of 0.975 (mean score, 28.98; standard deviation, 17.34). An exploratory factor analysis showed three factors and one factor with an eigenvalue > 1 that explained 75.176 and 62.83 % of the total variance in the Activity subscale and Participation subscale, respectively. The subscale-to-total scale correlation analysis showed Pearson correlation coefficients of 0.881 for the Activity subscale and 0.843 for the Participation subscale. Pearson’s product-moment correlation revealed that the correlation coefficient (*r*) between the Chinese version of the PaArticular Scales and the World Health Organization Disability Assessment Schedule was 0.770, whereas that for the World Health Organization Quality of Life scale was − 0.553; these values were interpreted as large coefficients.

**Conclusions:**

The underlying theoretical model of the Chinese version of the PaArticular Scales functions well in Taiwan and has acceptable levels of reliability and validity. However, the Chinese version must be further tested for applicability and generalizability in future studies, preferably with a larger sample and in different clinical domains.

**Supplementary Information:**

The online version contains supplementary material available at 10.1186/s12877-021-02297-5.

## Background

Joint contracture is a fibrous disease characterized by joint capsule fibrosis and a limited range of motion secondary to periarticular molecular connective tissue shortening [1]. Multiple factors are associated with joint contracture, the most important of which are joint incongruity, immobility, pain, adhesions, heterotopic bone formation, and periarticular connective tissue changes [2]. Interventions for joint contracture include surgical manipulation, botulinum toxin injection, splinting, joint mobilization, serial plaster application, electrical stimulation, and passive stretching [3].

Joint contractures are common complications of nervous system diseases and are problematic during rehabilitation [4]. Between 20 and 75 % of elderly residents in long-term care (LTC) facilities are affected by joint contractures [5], which result in functional restrictions and limitations of joint mobility and thus produce activity limitations and participation restrictions [5–7]. Many studies have noted that activity limitations and participation restrictions, such as the inability to write or visit friends, are most relevant to patients with joint contractures [8, 9].

Unfortunately, activity limitations and participation restrictions are closely related to the quality of life (QoL) of elderly residents in LTC facilities [10]. Many experts believe that QoL is an important outcome indicator for elderly residents in LTC facilities [11, 12]. Recent studies have examined the explanatory power of various factors for the QoL of elderly residents in LTC facilities and have found that activity and participation explain approximately 52 % of the variance in the QoL of this population [13]. This finding can help scholars and experts focus on the critical factors affecting the QoL of elderly residents in LTC facilities. Elderly individuals with joint contractures may have severely limited mobility, which could restrict their ability to participate in activities and negatively affect their QoL [8–10, 14].

Currently, the World Health Organization (WHO) Disability Assessment Schedule 2.0 (WHODAS 2.0) is a widely used scale for the global assessment of activity and participation; however, the scale has several issues. First, the population dynamics are heterogeneous with regard to the somatic and psychological symptoms of individuals, including those who are frail but still able to walk and individuals with severely constrained mobility. Second, the personal situations of individuals are diverse, including different LTC service needs and available resources. Third, the contracture categories and contracture sites of individuals differ; for example, individuals may have both or isolated upper and lower limb joint contractures. Fourth, activities and participation will vary in individuals with different categories and contracture sites [15].

The WHODAS 2.0, however, is designed to be applicable to all health conditions, including diseases, illnesses, injuries, mental or emotional problems, and alcohol or drug abuse. It does not attempt to assign aetiology or apportion impairment or disability to any particular disorder [16]. The evaluation of activity and participation is complicated, and the complex individual experience of impaired individuals must be recognized [15]. Therefore, an outcome questionnaire that quantifies the activity and participation of a particular population is especially important. To date, no universally accepted scale can address these key issues [15].

However, the International Classification of Functioning, Disability, and Health (ICF) is the common basis of the WHO’s patient-centred measures and intervention plan and comprehensively classifies all health and health-related fields [8]. Therefore, the PaArticular Scales, developed using the ICF as a standard, can fill in the gap. Furthermore, the validity and reliability of the PaArticular Scales has been documented in Germany (German version, Supplementary File 1) [8] and in the United States (English version, Supplementary File 2) [15]. To date, no nationwide survey has been conducted in Taiwan.

## Methods

### Aim

This study examined the reliability and validity of the Chinese version of the PaArticular Scales among elderly residents with joint contractures in LTC facilities.

### Design

This study was designed as a questionnaire-based cross-sectional survey.

### Settings and sample population

The multistage sampling method was used to perform random sampling between April and June 2020. For factor analysis, the sample size for items was 5 to 10 according to previous studies [17, 18]. The Chinese version of the PaArticular Scales (Supplementary File 3) has 35 items; therefore, a sample size of 175 to 350 participants was determined to be appropriate for factor analysis in this study [19].

The inclusion criteria of the participants were as follows: (1) age ≥ 65 years old; (2) residence at a facility for > 6 months; (3) sufficient language skills to complete the questionnaire; and (4) severe joint contractures in any single important joint (hand, elbow, shoulder, ankle, knee, or hip) with authentication from a doctor, nurse, or therapist. Severe joint contractures were defined by a score of 3 on a 4-point scale (loss of more than two-thirds of joint range of motion) [20, 21]. Individuals with cognitive impairment and major mental illness diagnosed by physicians were excluded.

### Study instrument

#### Demographic data

Levels of disability, classified as mild, moderate, severe, and extremely severe, were determined based on the Taiwan statutory LTC policies for older people. To further depict the personal characteristic data of the participants, the minimum data set (MDS) tool recommended on the interRAI country websites was used to record demographic data (such as education and religion) and the location and number of joint contractures (based on medical records and the MDS) [22].

#### Cognitive status

The Mini-Mental Status Examination (MMSE) was used to evaluate the cognitive status of participants [23]. This instrument employs a simple quantitative assessment scale and is widely used in clinics and research studies to evaluate cognitive function and screen cognitive impairment. The MMSE has 13 items, with a total score of 33, and takes only 5 to 10 min to complete. A higher score indicates better cognitive function. The test-retest reliability of the MMSE is good, and the interrater reliability correlation coefficient is 0.8 [23]. An MMSE score of 25 or less is defined as cognitive impairment [24].

#### Chinese version of the PaArticular Scales

The Chinese version of the PaArticular Scales for joint contractures was generated from the English version through five stages: translation, review, back-translation, review by a panel of specialists, and a pretest (Supplementary File 4). The Chinese version of the PaArticular Scales contains 35 items; the Activity subscale has 24 items, and the Participation subscale has 11 items. The sum of the Activity and Participation domain scores ranges from 0 (fewest limitations) to 47 (maximum limitations) and from 0 (fewest restrictions) to 19 (maximum restrictions), respectively. A higher score indicates greater activity limitations and participation restrictions. Because the scale is an organized face-to-face contact scale to assess activity and participation, before the assessment, the assessor emphasized that the study participants must describe their present surroundings and not imaginary surroundings or their original home surroundings.

In terms of reliability, Cronbach’s *α* coefficients of the internal consistency of the Activity subscale and the Participation subscale are 0.96 and 0.92, respectively, in the English version. Additionally, McDonald’s *ω* totals are 0.98 and 0.95, respectively [15], indicating high internal consistency. The Pearson correlation coefficients of the Activity and Participation subscales determined using the criterion validity of the visual analogue scale of the EuroQol-5 dimensions (EQ-5D), which is one of the most frequently used generic health status measurement tools, demonstrated good validity and reliability, at − 0.40 (*p* > .001) and − 0.30 (*p* > .001), respectively [15]. The EQ-5D is a generic health-related QoL questionnaire [25]. The Activities and Participation subscales have negative Pearson correlations with the EQ-5D, indicating that higher QoL corresponds to lower activity limitations and participation restrictions.

#### The WHO Quality of Life (WHOQoL)-BREF

The short version of the WHO’s Quality of Life Instrument (WHOQoL-BREF) is widely validated and popularly used to assess the subjective QoL of patients and the general public [26]. To evaluate the criterion validity of the Chinese version of the PaArticular Scales, we used the Chinese version of the WHOQoL-BREF developed from the WHO’s WHOQoL group, which contains 26 items (Supplementary File 5). When the amount of missing data is large (greater than 10 %), the results of subsequent statistical analyses may be biased [27]. Therefore, questionnaires with more than 20 % missing data were discarded. Multiple imputation methods were used to manage missing data. If more than two values were missing in a domain, the domain score was not calculated (except for domain 3; the score was calculated only if the missing value was < 1).

For the Chinese version of the WHOQOL-BREF, Cronbach’s *α* coefficient for the internal consistency of the overall questionnaire ranged from 0.73 to 0.83, and the test-retest reliability coefficients of each category ranged from 0.41 to 0.79 [28]. The Pearson correlation between each item and its category ranged from 0.45 to 0.82 (*p* < .01), and the correlation between different categories ranged from 0.48 to 0.63 (*p* < .01). For the confirmatory factor analysis (CFA) of the construct validity, the structural equation model of the four factors replicates the potential structure designed by the questionnaire, and the comparative fitness indices (CFIs) of these two analyses had values of 0.886, which is equivalent to that of the Hong Kong version of the questionnaire (CFI = 0.894) and similar to that of the questionnaire using global data (CFI = 0.903) [28].

#### The WHO Disability Assessment Schedule 2.0 (WHODAS 2.0)-36 items

To evaluate the criterion validity of the Chinese version of the PaArticular Scales, we used the Chinese version of the WHODAS 2.0–36 items. Participants used a 5-point Likert scale to answer questions related to difficulties in performing activities. The score ranged from 0 (least difficulty) to 100 (maximum difficulty) and was calculated as the sum of each domain score [29]. A higher score indicated a higher degree of disability and more severe restriction. Restriction severity refers to the difficulty level classification method of the ICF and WHODAS 2.0. The classification of impairment severity was as follows: below 4 %, none; 5–24 %, mild; 25–49 %, moderate; 50–95 %, severe; and more than 96 %, extremely severe [30]. Four items in the WHODAS 2.0 cover job domains (e.g., Have you ever had to accept a lower-level job because of health factors?); thus, only 32 items were calculated in the study because all participants were retired and unemployed.

Regarding the reliability indices of the Chinese version of the WHODAS 2.0, Cronbach’s *α* for internal consistency is between 0.70 and 0.99, and the intraclass correlation coefficient is between 0.80 and 0.89 [31, 32]. Among the validity indices, the content and the concurrent validity have some correlation, and based on exploratory factor analyses (EFAs), 5 to 7 factors have an explanatory power higher than 55 %. The factor loadings of the CFA are all higher than 0.56 [31]. Therefore, the Chinese version of the WHODAS 2.0 has excellent reliability and validity and is consistent with item response theory. In the current study, authorities have granted permission for the use of each Chinese version of the survey instrument .

### Data collection procedure

The resident list was obtained approximately 1 week after communicating with the LTC facilities, and institutional residents who met the criteria were selected from the list. The study participants generally completed the questionnaire independently. However, if a participant was unable to complete the questionnaire independently because of vision, hearing, reading, or writing limitations, then the interviewers trained in the consensus camp provided assistance (e.g., explaining some sentences to clarify the meaning). Three interviewers were trained in basic counselling techniques and relaxation exercises and were trained to continue until a consensus was reached on the meaning and means of the questionnaires. Care was taken that the method of assistance provided by the interviewers in answering the questions was consistent for all of the participants (e.g., the examples provided for answering the questions were the same), which followed the data collection procedure of Chen et al. [13].

### Data analysis

During survey completion, some data were not collected due to refusal of the respondents, negligence of the investigators, or issues with the questionnaire itself but were resolved by multiple imputation methods. The goal of using descriptive statistics was to gain an understanding from the personal characteristic data of the participants. Absolute and relative frequencies were determined for categorical data, and the mean and standard deviation (SD) were determined for continuous data.

#### Test-retest reliability

The current study determined the test-retest reliability of the Chinese version of the scale by evaluating participants. Residents were revisited 3 days later by a different assessor who they had not met previously, and the scale was assessed again. Using the comprehensive Cohen κ statistic, including 95 % confidence intervals, the personal items and the administrator rating were compared under continuous testing. The κ value is expressed as a number between 0 and 1, where 0 indicates no agreement and 1 indicates complete agreement; therefore, κ values of ≥ 0.81 indicate almost perfect agreement, those between 0.80 and 0.61 indicate substantial agreement, and those between 0.60 and 0.41 indicate moderate agreement [33].

#### Internal consistency reliability

The internal consistency reliability was tested by using McDonald’s *ω* (hierarchical), Cronbach’s *α*, and McDonald’s *ω* total for each competency [34]. The *α* value is expressed as a number between 0 and 1; thus, an *α* value of ≥ 0.91 indicates excellent reliability, and a value between 0.90 and 0.71 indicates acceptable reliability [35]. Two types of item analysis were used, including (1) within-item relevance and (2) item-to-total correlation, to analyse the homogeneity of the research tool. Finally, the correlation between the subscale and the total scale was analysed. Cronbach’s *α* coefficient was used to measure the internal consistency reliability between the Chinese version of the PaArticular Scales and its subscales.

#### Construct validity

The Kaiser-Meyer-Olkin (KMO) and Bartlett sphericity tests were both performed to determine whether the data collected from the questionnaire were suitable for factor analysis. According to the principle of varimax rotation, EFA was used to assess the validity of the Chinese version of the PaArticular Scales. The original English version of the PaArticular Scales has good criterion validity and internal consistency reliability [15]. EFA was used to determine the essential structure of multivariate observations. The factors were first selected based on a screening index of an eigenvalue > 1.0 [36]. The factors were selected again based on a scree plot, clinical experience, and the original factor structure of the scales [37–40]. Finally, the items were selected, provided that the minimum variance of each factor was 5 %.

#### Criterion validity

Convergent construct validity was evaluated by comparing the Pearson correlation coefficients of the Chinese version of the PaArticular Scales to both the WHOQoL-BREF and WHODAS 2.0–36 items, which have been used previously among elderly residents in LTC facilities. These comparisons are valuable because alterations in activity and participation are accompanied by alterations in participants’ QoL [41, 42]. The point-biserial correlation coefficient was used to calculate the correlations among the Chinese version of the WHOQoL-BREF, the Chinese version of the WHODAS 2.0–36 items, and the Chinese version of the PaArticular Scales total score to establish concurrent validity. All data were statistically analysed using the SPSS 22.0 software package (IBM, Armonk, NY, USA).

## Results

In this study, we randomly sampled 300 participants from 12 LTC facilities who met the inclusion criteria; eight individuals were unwilling to complete the consent form, and 49 were unwilling to complete the questionnaire due to emotional factors. Finally, a total of 243 participants were included in the data analysis. The power (1 − *β*) was 0.99 at a 95 % confidence level with G-power program tests in the domain of correlation analyses when applying an effect size of 0.50, *α* = 0.05. Five participants completed the questionnaire after receiving explanations of the questions. Among the participants, 14.81 % (*n* = 36) had upper extremity contractures, 64.20 % (*n* = 156) had lower extremity contractures, and 21.99 % (*n* = 51) had both upper and lower extremity contractures. The demographic characteristics of the participants are provided in Table 1.

**Table 1:** Descriptive characteristics of the participants (*N* = 243)

Characteristics	Mean		*SD*		Min–max
Number of joint contractures	1.94		1.22		1–6
Age (years)	72.38		11.91		65–97
Length of residency (months)	38.26		46.36		1–240
Body mass index (kg/m^2^)	22.33		3.42		12.12–34.73
Characteristics		n		%	
Contracture sites					
Upper limbs		36		14.81	
Lower limbs		156		64.20	
Upper and lower limbs		51		21.99	
Gender					
Male		156		64.20	
Female		87		35.80	
Ancestry/ethnicity					
Min Nan		228		93.83	
Hakka		9		3.70	
Aboriginal		3		1.23	
Mainland Chinese		3		1.23	
Education					
Primary and below		144		59.26	
Junior high		57		23.46	
Senior secondary		24		9.88	
Higher		9		3.70	
College/university and above		9		3.70	
Occupation					
Military		0		0	
Management		6		2.47	
Professional		18		7.41	
Technical activities and assistant professional		0		0	
Office and administrative support		36		14.81	
Sales and service		0		0	
Agriculture, forestry, fishing, and animal husbandry		12		4.94	
Arts, design, entertainment, sports, and media		57		23.46	
Installation, maintenance, and repair		12		4.94	
Production and labourers		69		28.40	
Unemployed		33		13.58	
Marital status					
Married		57		23.46	
Widowed		69		28.40	
Divorced		33		13.58	
Single		84		34.57	
Religion					
Buddhism		60		24.69	
Taoism		138		56.79	
Christianity/Catholicism		24		9.88	
Atheist		15		6.17	
Others		6		2.47	
Visitation frequency (weeks)					
0–1		174		71.60	
2–3		54		22.22	
4–5		9		3.70	
6–7		6		2.47	
Chronic diseases					
Hypertension		75		30.86	
Diabetes		60		24.69	
Stroke		63		25.93	
Cardiovascular disease		36		14.81	
Cancer		0		0	
Degenerative arthritis		54		22.22	
Cataract		3		1.23	
Glaucoma		3		1.23	

*SD* Standard deviation

### Research flow chart

**Fig. 1:**
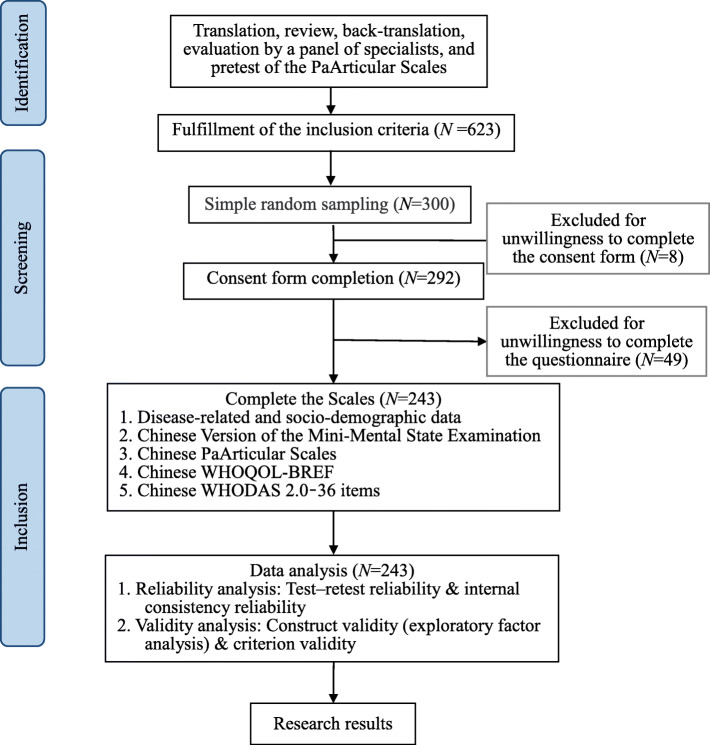
The “Strengthening the Reporting of OBservational Studies in Epidemiology” (STROBE) Diagram: Flow of Participants Throughout the Study. WHODAS 2.0–36 items: The WHO Disability Assessment Schedule 2.0–36 items; WHOQoL-BREF: The World Health Organization Quality of Life-BREF

A flow chart of this study is shown in Fig. 1, together with the instruments administered, sampling procedures, and number of responses for each sample.

### Reliability test

Cronbach’s *α* of the Activity subscale, which consisted of 24 items, was 0.97, with an average score of 18.68 (SD, 13.51). Cronbach’s *α* coefficient of the Participation subscale, which consisted of 11 items, was 0.94, with an average score of 10.30 (SD, 5.85). Therefore, both subscales had excellent internal consistency. The 35-item Chinese version of the PaArticular Scales had a Cronbach *α* coefficient of 0.98 and an average score of 28.98 (SD, 17.34), indicating that this scale had excellent reliability (Table 2). The subscale-to-total scale correlation analysis showed Pearson correlation coefficients of 0.881 for the Activity subscale and 0.843 for the Participation subscale.

**Table 2:** Descriptive statistics and Cronbach’s *α* coefficients (*N* = 243)

Subscale	Number of items	Mean (SD)	Cronbach’s *α*
Activities scale	24	18.68 (13.51)	0.97
Participation scale	11	10.30 (5.85)	0.94
PaArticular Scales total	35	28.98 (17.34)	0.98

### Validity testing

The KMO and Bartlett sphericity tests were both performed. The test results showed that the KMO values of the Activity subscale and Participation subscale were 0.91 and 0.89, respectively; KMO values > 0.50 indicate common factors in the questionnaire items (i.e., the factors are independent). The results of Bartlett’s χ^2^ test were all statistically significant (*p* < .001), indicating that the factors were independent and exclusive. Therefore, the questionnaire data collected in this study were suitable for factor analysis.

EFA was used to extract the construct of the scale. The Activity subscale had three factors with an eigenvalue > 1, explaining 75.18 % of the total variance in the scale. However, according to the scree plot, the curve flattened after the fourth factor. Therefore, based on the standard and scree plots for the factors with eigenvalues > 1.00 and combined with clinical experience and the factorial structure of the raw scores [37–40, 43], we postulated that the Activity subscale of the Chinese version had three main factors: lower limb activity, upper limb activity, and self-care activity. One factor with an eigenvalue > 1.00 was obtained from the participation subscale, explaining 62.83 % of the total variance in the scale. However, according to the scree plot, the curve flattened after the second factor. The Participation subscale of the Chinese version had only one major factor: participation. Tables 3 and 4 show the factor structure after rotation of the Activity and Participation subscales.

**Table 3:** Item loadings for the Chinese version of the Activity Subscale

Item No.	Item	Factor 1: lower limbs	Factor 2: upper limbs	Factor 3: self-care
2	Stand up and change positions (e.g., sit down again from a standing position)	0.84	0.30	0.19
1	Stand up or sit down	0.82	0.28	0.26
3	Sit down or stand up as long as you need to	0.81	0.31	0.20
6	Slide from one bed to another independently or relying on a shifting board	0.74	0.38	0.36
4	Stop as long as you need to	0.73	0.01	0.48
5	Move from one seat to another without getting up completely	0.68	0.24	0.46
15	Move between the rooms in your apartment and adjacent hallways on the same floor	0.64	0.32	0.43
7	Carry or lift things from one place to another	0.60	0.57	0.35
16	Walk and move around inside unknown or unrecognized architectures	0.59	0.25	0.58
18	Use a privately owned automobile or cab as a mobile tool	0.55	0.20	0.54
9	Pick up a small object with hands and fingers (e.g., lifting a brush)	0.27	0.87	0.22
8	Make fine-tuning movements with hands (such as painting, closing buttons or tying shoelaces)	0.27	0.85	0.20
13	Pull an object towards you or push it away with your arms, hands and fingers (e.g., closing a gate or mosquito net or pushing away a table)	0.16	0.79	0.35
14	Use your fingers, hands and arms to twist (water) bottle caps or tie a pinafore or turn lock openers	0.32	0.79	0.22
12	Intentionally let go of things in your hands	0.10	0.77	0.43
10	Hold an object with both hands, such as holding a tackle	0.46	0.75	0.19
11	Take out money from an open wallet in the store to make a purchase	0.38	0.61	0.41
23	Independent diet-related activity (including opening cans, bagging, caps and the use of knives, spoons, forks)	0.24	0.56	0.54
21	Use the toilet (such as going to the shower room, putting on and taking off your clothes/diapers, or taking a bath)	0.38	0.29	0.76
24	Take care of your well-being and health independently (such as physical activity, use of medication, appointments for outpatient clinics)	0.30	0.32	0.75
17	Do you have difficulties moving independently with aids (e.g., wheelchair, walker, or stick) around the house or outside?	0.42	0.29	0.67
20	Look after your appearance and perform personal hygiene such as taking care of your nails, teeth, genitals, skin, face, and scalp	0.22	0.46	0.67
22	Undress from head to toe independently (e.g., zipping, tying belts and handling buttons, removing clothes)	0.32	0.35	0.66
19	Use public transport as a passenger, e.g., plane, train or bus	0.41	0.31	0.52
% of variance Cumulative % Eigenvalue		26.78	26.20	22.20
		26.78	52.98	75.18
		14.82	2.07	1.15

**Table 4:** Item loadings for the Chinese version of the Participation Subscale

Item No.	Item	Factor 1: participation
4	Participate in community life on the basis of your expectations	0.89
11	Take part in religious belief activities	0.85
5	Take part in games (such as board, memory, card games)	0.84
10	Attend social gatherings	0.82
9	Run after your hobbies	0.81
7	Engage in your cultural hobbies	0.81
6	Take part in gymnastics and sports activities	0.80
2	Use appropriate physical touch with other people (such as hugging in accordance with appropriate social etiquette)	0.79
3	Communicate with others and keep going social contacts	0.75
1	In various fields of daily life, help others in need	0.68
8	Tinker/handicraft	0.64
% of variance Cumulative % Eigenvalue		62.83
		62.83
		6.91

The correlation coefficients for factors 1, 2, and 3 and the item-to-subscale were obtained to determine content validity; the ranges for these coefficients were 0.73 to 0.89, 0.71 to 0.89, and 0.62 to 0.85, respectively, and Cronbach’s *α* values were 0.96, 0.95, and 0.91, respectively.

The criterion-related validity was determined according to the classification proposed by Cohen [44], and Pearson’s product-moment correlation revealed that the correlation coefficient (*r*) between the Chinese version of the PaArticular Scales and the WHODAS 2.0–36 items was 0.77, which was interpreted as a large coefficient, with a *p*-value of less than 0.001, indicating a highly significant result. The correlation coefficient (*r*) between the Chinese version of the PaArticular Scales and the WHOQoL-BREF was − 0.55, which was interpreted as a large coefficient, with a *p*-value of less than 0.001, indicating a highly significant result. The negative correlation between the PaArticular Scales and the WHOQoL-BREF indicates that higher QoL corresponds to lower activity limitations and participation restrictions. The correlation coefficients between the Activity subscale and the WHODAS 2.0–36 item and WHOQoL-BREF were 0.72 and − 0.50, respectively, and the correlation coefficients between the Participation subscale and the WHODAS 2.0–36 item and WHOQoL-BREF were 0.74 and − 0.58, respectively; all correlation coefficients were highly significant (Table 5).

**Table 5:** Correlations between the Chinese version of the PaArticular Scales and the WHODAS 2.0–36 items and WHOQoL-BREF (*N* = 243)

	WHODAS 2.0	WHOQoL-BREF
PaArticular Scales Total	0.77**	−0.55**
Activity Subscale	0.72**	−0.50**
Factor 1: lower limbs	0.73**	−0.43**
Factor 2: upper limbs	0.52**	−0.45**
Factor 3: self-care	0.74**	−0.53**
Participation Subscale	0.74**	−0.58**
Factor 1: participation	0.74**	−0.58**

**p < .01 (two-tailed). *WHODAS 2.0* The WHO Disability Assessment Schedule; *WHOQoL-BREF* The WHO Quality of Life Scale Abbreviated Version

## Discussion

Most participants were able to complete the questionnaire without assistance, and only five participants were unable to complete the questionnaire because they were unable to understand the questions. When a participant cannot correctly complete a questionnaire independently, additional risks to validity may be unavoidable. Such risks may stem from inaccurate data collection, interviewer effects, or changes in the research procedure over time, or an interviewer may begin an interview with expectations that preclude solicitation of relevant information to inform rating judgements [45], all of which may compromise the validity of interviewer reports and self-reports. In addition, we ensured that the researchers provided assistance consistently to all the participants when answering the questions, which followed the data collection procedure of Chen et al. [13]. We did not check the data after the patients completed the questionnaire.

The reliability of the outcome of the Chinese version of the PaArticular Scales assessment tool was verified in Taiwan and satisfied the item analysis for items associated with the validity. This study found that the Activity subscale had three factors (i.e., latent variables): lower limb activity, upper limb activity, and self-care activity. The Participation subscale had a single factor, participation. The two subscales explained 75.18 and 62.83 % of the variance of the scale, respectively, indicating that the results had practical significance.

The Chinese version of the PaArticular Scales had excellent internal consistency and reliability. Cronbach’s *α* coefficients for the Activity subscale and Participation subscale were 0.97 and 0.94, respectively, which were slightly higher than those found by Müller et al. [15] for 191 elderly residents with joint contractures in German LTC facilities (*α* = 0.96 and 0.92). Although the two studies were performed in different countries, the *α* coefficients were very similar. According to the standard set by Nunnally and Bernstein (*α* coefficient ≥ 0.80) [46], the PaArticular Scales have excellent internal consistency and reliability across ethnic groups.

The criterion validity tests showed that for individuals older than 64 years with severe joint contractures, strong evidence indicates that the Chinese version of the PaArticular Scales is linearly related to the WHODAS 2.0–36 items (*r* = .77, *p* < .001). Furthermore, the Pearson correlation coefficient was large. These results show that, similar to the WHODAS 2.0–36 items, the PaArticular Scales developed using the ICF of the WHO as the standard can be used as another simple tool for clinical measurement of activity and participation, and this tool addresses the gap in assessing patients with joint contractures [8]. However, although the Chinese version of the PaArticular Scales is also based on the ICF, it is mainly used for patients with joint contractures, which is different from the widely used WHODAS 2.0–36 items. This difference might explain why the correlation between the two scales was not very high. Another reason may be that most of the participants in this study were institutionalized residents and required nursing care. These characteristics are clearly not considered to be associated with the applicable subjects for the WHODAS 2.0–36 items; therefore, the results may be attributable to many different characteristics of the participants, for example, physical conditions.

Criterion validity was also assessed to test the correlation between the Chinese version of the PaArticular Scales and the established Chinese version of the WHOQoL-BREF. For individuals older than 64 years with severe joint contractures, very strong evidence indicates that the Chinese version of the PaArticular Scales is linearly related to the WHOQoL-BREF (*r* = − .55, *p* < .001). Furthermore, the Pearson correlation coefficient was large. The newly developed scale demonstrated criterion validity, which was consistent with findings by Chen et al. [13]. The results showed that activity and participation, personal factors, and body function and structure are determinants of QoL for elderly residents in LTC facilities. Among them, activity and participation have the greatest explanatory power, up to 52 %, indicating that activity and participation have practical value for the QoL of elderly residents. The results also echo the view of Rantanen et al. [14] in that providing outdoor activities for elderly residents with severely limited mobility may positively affect their QoL.

Notably, although the published study of the original scale mentioned the use of factor analysis and briefly described the analysis process [15], it did not disclose clear analysis results and data. The purpose of applying factor analysis is to exclude unsuitable items. After some steps, many items showed sufficient model fit, and we decided to build a separate scale for activities and regarded the remaining items from the original set as candidates for a separate scale for participation. Finally, the initial items were classified into two subscales: Activity and Participation. The part of the scale developed in the present study that is superior to the original scale published in the literature is the detailed analytical data organized into a table. The two subscales were further analysed, and the study results showed that the Activity subscale has three factors, and the Participation subscale has one factor. Moreover, the present study provides topic names based on the characteristics of the items. As this is a newly developed scale, available data are limited. Therefore, discussions regarding factor analysis of the scale will be left for future studies, and then, further discussions and analyses of this issue can be conducted.

Some potential limitations should be considered. First, the data in this study were obtained from a self-report questionnaire. Although most of the responses were fully validated, predicting or estimating the subjective bias of reported data is still difficult. For example, in the analysis of the reported data, deviations in the actual experience of the participants might exist.

Second, the participants were recruited from LTC facilities, and the design considerations of this study could only reflect the view of the included ethnic groups. Although demographic variables, such as the participants’ age, gender, education, and visitation rate, were controlled, caution should be used when generalizing these findings to other settings or to other elderly populations.

Third, although the sample size of this study satisfied the requirements for establishing stable person and item estimates and power analysis [19], the Chinese version of the PaArticular Scales must be studied with a larger sample size to obtain more complete and reliable data.

Finally, LTC residents with cognitive impairment were excluded because they may not have been able to complete the scale correctly (e.g., biases in memory and beliefs). However, such exclusion may have been too extensive to detect the prevalence of the study effects because cognitive impairment is very common in LTC residents and frequently coexists with joint contractures.

The limitations complicate interpretation of the results and their application to joint contractures in the cognitive impairment group. Therefore, the Chinese version must be further tested in future studies for applicability and generalizability, preferably in a larger sample, in cognitive impairment populations, and in different clinical domains.

## Conclusions

This study demonstrated that the Chinese version of the PaArticular Scales is a reliable and effective tool for measuring the activity and participation of elderly individuals with joint contractures. As a sound outcome measurement tool, the Chinese version of the PaArticular Scales developed in this study not only fills the gap in assessing the activity and participation of elderly Chinese individuals but also makes the evaluation of elderly individuals with joint contractures more comprehensive, which can provide a basis for improving their activity, participation, and QoL. Furthermore, this tool can be used in the treatment, rehabilitation, prevention, and research programmes of LTC facilities.

## Supplementary Information

**Additional file 1:**

**Additional file 2:**

**Additional file 3:**

**Additional file 4:**

**Additional file 5:**

## Data Availability

The data sets generated and analysed during the current study are available in the Chinese Clinical Trial Registry (ChiCTR2000030413) repository, registered 1 March 2020, http://www.chictr.org.cn/usercenter.aspx.

## Abbreviations

CFA: Confirmatory factor analysis
CFI: Comparative fitness index
EFAs: Exploratory factor analyses
EQ-5D: EuroQol-five dimensions
ICF: International Classification of Functioning, Disability, and Health
KMO: Kaiser-Meyer-Olkin
LTC: Long-term care
MDS: Minimum data set
MMSE: Mini-Mental Status Examination
QoL: Quality of life
SD: Standard deviation
WHODAS 2.0: WHO Disability Assessment Schedule 2.0
WHOQoL: World Health Organization Quality of Life
