# Supplementary material for: Psychometric validation of the Chinese version of the PaArticular Scales among elderly residents in long-term care facilities with joint contractures
Source: BMC Geriatr. 2021 Jun 9;21:353. doi: 10.1186/s12877-021-02297-5 (PMC8190856; doi:10.1186/s12877-021-02297-5)
Supplement: Supplementary file 1 — Additional file 1: [file 12877_2021_2297_MOESM1_ESM.pdf]

Aktivitätenskala

	Haben Sie Schwierigkeiten, …	Keine (0-4%)	Leichte (5-49%)	Mäßige (50-95%)	Erhebliche (96-100%)
		Einschränkung			
d4103	sich hinzusetzen oder aufzustehen?	0	1	1	2
d4104	sich hinzustellen und sich dann aus dem Stand wieder hinzulegen oder hinzusetzen?	0	1	1	2
d415	so lange zu stehen oder zu sitzen wie es für Sie nötig ist?	0	1	1	2
d4154	solange stehen zu bleiben, wie es für Sie nötig ist?	0	1	1	2
d4200	von einer Sitzgelegenheit auf eine andere zu wechseln, ohne dabei ganz aufzustehen?	0	1	1	2
d4201	von einem Bett in ein anderes oder auf eine Transportliege zu rutschen?	0	1	1	2
d430	einen Gegenstand anzuheben oder etwas von einem Platz zu einem anderen zu tragen?	0	1	1	2
d440	bei der Feinkoordination Ihrer Hände z.B. beim Schreiben, Knöpfe zumachen oder Binden der Schuhe?	0	1	1	2
d4400	einen kleinen Gegenstand, wie z.B. einen Bleistift mit den Händen und Fingern aufzunehmen oder aufzuheben?	0	1	1	2
d4401	einen Gegenstand mit beiden Händen zu ergreifen und zu halten, wie z.B. ein Werkzeug?	0	1	1	2
d4402	Geld aus einer geöffneten Brieftasche zu entnehmen, um damit in einem Geschäft zu bezahlen?	0	1	1	2
d4403	einen Gegenstand absichtlich loszulassen?	0	1	1	2
d4450	einen Gegenstand mit Fingern, Händen und Armen zu sich heranzuziehen oder wegzuschieben, wie eine Tür oder Gardine zuzuziehen oder einen Stuhl wegschieben?	0	1	1	2

d4453	mit Ihren Fingern, Händen und Armen eine (Wasser)Flasche aufzuschrauben, eine Schürze zu binden oder einen Schlüssel zu drehen?	0	1	1	2
d4600	sich zwischen den Zimmern in Ihrer Wohnung und dem anschließenden Gang auf dem gleichen Stockwerk umherzubewegen?	0	1	1	2
d4601	sich innerhalb fremder Gebäude umherzubewegen?	0	1	1	2
d465	Haben Sie Schwierigkeiten sich selbstständig mit Hilfsmitteln wie Stock, Rollator oder Rollstuhl, sowohl innerhalb als auch außerhalb fortzubewegen?	0	1	1	2
d4701	als Fahrgast ein privates Auto oder ein Taxi zu benutzen?	0	1	1	2
d4702	als Fahrgast ein öffentliches Verkehrsmittel z.B. Bus, Bahn oder Flugzeug zu benutzen?	0	1	1	1
d520	Ihr Erscheinungsbild zu pflegen und Ihre Körperpflege durchzuführen, wie Haut, Gesicht, Zähne, Kopfhaut, Nägel und Genitalien zu pflegen?	0	1	1	2
d5300	mit dem Toilettengang (z.B. beim Aufsuchen der Toilette, beim Ausziehen und wieder Anziehen, beim Abwischen oder bei der Benutzung von Inkontinenzhilfsmitteln)?	0	1	1	2
d540	sich selbstständig von Kopf bis Fuß ihre Kleidung (z.B. Entnehmen der Kleidung, Handhabung von Knöpfen und Reißverschlüssen, Schleifen binden) an- und auszuziehen?	0	1	1	2
d550	selbstständig zu essen (einschließlich Benutzung von Messer, Gabel, Löffel, sowie Öffnen von Flaschen, Dosen, Verpackungen)?	0	1	1	2
d570	selbstständig für ihr Wohlbefinden und ihre Gesundheit zu sorgen (z.B. Medikamentengebrauch, körperliche Aktivität, Arztbesuch)?	0	1	1	2

©Ludwig-Maximilians-Universität München und Martin-Luther-Universität Halle-Wittenberg

Partizipationsskala

		Keine (0-4%)	Leichte (5-49%)	Mäßige (50-95%)	Erhebliche (96-100%)
	Haben Sie Schwierigkeiten, …	Einschränkung			
d660	hilfsbedürftige Menschen in verschiedenen Bereichen des täglichen Lebens zu unterstützen?	0	1	1	1
d7105	in angemessener Weise mit anderen Menschen körperlichen Kontakt aufzunehmen (z.B. jemanden bei der Begrüßung zu umarmen)?	0	1	1	2
d750	mit anderen Menschen soziale Kontakte aufzunehmen und zu pflegen?	0	1	1	2
d910	entsprechend Ihren Vorstellungen am gesellschaftlichen Leben teilzunehmen?	0	1	1	2
d9200	sich an Spielen (z.B. Kartenspiele, Memory, Brettspiele) zu beteiligen?	0	1	1	2
d9201	sich an Gymnastik und sportlichen Aktivitäten zu beteiligen?	0	1	1	2
d9202	Ihre kulturellen Interessen zu verwirklichen?	0	1	1	1
d9203	zu basteln/zu handarbeiten?	0	1	1	1
d9204	Ihren Hobbies nachzugehen?	0	1	1	2
d9205	an geselligen Zusammenkünften teilzunehmen?	0	1	1	2
d930	Ihre Religiosität auszuüben?	0	1	1	2

©Ludwig-Maximilians-Universität München und Martin-Luther-Universität Halle-Wittenberg
