# Supplementary material for: Psychometric validation of the Chinese version of the PaArticular Scales among elderly residents in long-term care facilities with joint contractures
Source: BMC Geriatr. 2021 Jun 9;21:353. doi: 10.1186/s12877-021-02297-5 (PMC8190856; doi:10.1186/s12877-021-02297-5)
Supplement: Supplementary file 2 — Additional file 2: [file 12877_2021_2297_MOESM2_ESM.pdf]

Activities scale

Do you have any problems/difficulties, …	No (0-4%)	Mild/moderate (5-49%)	Severe (50-95%)	Full (96-100%)
Sitting down or standing up?	0	1	1	2
Standing up and getting out of a standing position to another position, such as lying down or sitting down?	0	1	1	2
Standing or sitting as long as you need to?	0	1	1	2
Staying in a standing position for some time as required?	0	1	1	2
Moving from a sitting position on one seat to another seat without standing up completely?	0	1	1	2
Sliding from one bed to another or to a stretcher for transport?	0	1	1	2
Lifting an object or carrying something from one place to another?	0	1	1	2
With fine coordination of your hands, e.g. writing, buttoning or lacing your shoes?	0	1	1	2
Lifting a small object with your hands and fingers, such as picking up a pencil?	0	1	1	2
Grasping and holding an object using both hands, e.g. grasping a tool?	0	1	1	2
Taking money out of an open purse in order to pay in a shop?	0	1	1	2
Releasing an object on purpose?	0	1	1	2
Using fingers, hands and arms to bring an object towards yourself, or move it away from yourself, such as when closing a door or a curtain or pushing a chair aside?	0	1	1	2

Unscrewing a bottle cap or tying an apron or turning a key with your fingers, hands and arms?	0	1	1	2
Moving around between rooms and the adjacent corridor on the same floor within your home?	0	1	1	2
Walking and moving around within unfamiliar buildings?	0	1	1	2
Moving around using equipment, such as a walking stick, a walker or wheelchair both inside and outside.	0	1	1	2
Using private car or a taxi as a passenger?	0	1	1	2
Using public transportation, such as being passenger on a bus, train or aircraft?	0	1	1	1
Maintain your appearance and caring for body parts, such as skin, face, teeth, scalp, nails and genitals?	0	1	1	2
With toileting (e.g. visiting the bathroom, manipulating clothing before and after, cleaning yourself or using incontinence aids)?	0	1	1	2
Putting on and taking off clothes from head to foot without assistance (e.g. picking clothes, doing buttons and zippers, tying a bow)?	0	1	1	2
Eating autonomously (including the use of fork, knife, spoon, as well as opening bottles, cans, packages)	0	1	1	2
Autonomously taking care of your wellbeing and health (e.g. managing daily medications, physical activity, scheduling doctor´s appointments)	0	1	1	2

©Ludwig-Maximilians-Universität München and Martin-Luther-Universität Halle-Wittenberg reproduced/translated with kind permission of the copyright owners

Participation scale

Do you have any problems/difficulties, …	No (0-4%)	Mild/moderate (5-49%)	Severe (50-95%)	Fiull (96-100%)
Supporting people who need assistance in different areas of daily life?	0	1	1	1
Bodily contacting others in a contextually and socially appropriate manner (e.g. hugging someone as a greeting)?	0	1	1	2
Engaging and maintaining social relationships with other people?	0	1	1	2
Taking part in community life according to your expectations?	0	1	1	2
Participating in games (e.g. playing cards, memory games, board games)?	0	1	1	2
Participating in exercise and sports?	0	1	1	2
Realizing your cultural interests?	0	1	1	1
Doing handycraft or needlework?	0	1	1	1
Pursuing your hobbies?	0	1	1	2
Taking part in social gatherings / getting together with friends or relatives?	0	1	1	2
Practicing your religion?	0	1	1	2

©Ludwig-Maximilians-Universität München and Martin-Luther-Universität Halle-Wittenberg reproduced/translated with kind permission of the copyright owners
