# Supplementary material for: Psychometric validation of the Chinese version of the PaArticular Scales among elderly residents in long-term care facilities with joint contractures
Source: BMC Geriatr. 2021 Jun 9;21:353. doi: 10.1186/s12877-021-02297-5 (PMC8190856; doi:10.1186/s12877-021-02297-5)
Supplement: Supplementary file 3 — Additional file 3: [file 12877_2021_2297_MOESM3_ESM.docx]

**活動量表(Activities scale)**

|  | 您有什麼問題/困難嗎？ | 沒有問題  (0-4%) | 輕/中度  (5-49%) | 重度  (50-95%) | 完全困難  (96-100%) |
| --- | --- | --- | --- | --- | --- |
| 1 | 坐下或站起來？ | 0 | 1 | 1 | 2 |
| 2 | 站起來且轉換不同姿勢﹙如躺下或坐下﹚？ | 0 | 1 | 1 | 2 |
| 3 | 視需要，站起來或坐下？ | 0 | 1 | 1 | 2 |
| 4 | 視需要保持一段時間的站姿？ | 0 | 1 | 1 | 2 |
| 5 | 在不完全站直情況下，從一個座位移動到另一個座位？ | 0 | 1 | 1 | 2 |
| 6 | 獨立或依靠移位板，從一張床滑到另一張床？ | 0 | 1 | 1 | 2 |
| 7 | 提或攜帶物品從一個地方到另一個地方？ | 0 | 1 | 1 | 2 |
| 8 | 用手作精細動作﹙如寫字、釦鈕扣或繫鞋帶﹚？ | 0 | 1 | 1 | 2 |
| 9 | 用手和手指舉起一個小物體﹙如拿起一支鉛筆﹚？ | 0 | 1 | 1 | 2 |
| 10 | 用雙手握住物體，如握住一個工具？ | 0 | 1 | 1 | 2 |
| 11 | 在商店裡從開放式的錢包拿出錢付費？ | 0 | 1 | 1 | 2 |
| 12 | 有目的的放掉手中東西？ | 0 | 1 | 1 | 2 |
| 13 | 用手指、手和手臂將物體帶到身邊或從身體移開﹙如關門或窗簾或將椅子推到一邊﹚？ | 0 | 1 | 1 | 2 |
| 14 | 用手指、手和手臂擰瓶蓋或綁圍裙或轉鑰匙？ | 0 | 1 | 1 | 2 |
| 15 | 在同一層樓內，房間和相鄰的走廊之間移動？ | 0 | 1 | 1 | 2 |
| 16 | 在不熟悉的建築物內散步和移動？ | 0 | 1 | 1 | 2 |
| 17 | 利用輔具(如手杖、助行器或輪椅)在屋裡屋外移動？ | 0 | 1 | 1 | 2 |
| 18 | 使用私家車或出租車作為移動工具？ | 0 | 1 | 1 | 2 |
| 19 | 使用公共交通工具﹙如乘坐公共汽車、火車或飛機﹚？ | 0 | 1 | 1 | 1 |
| 20 | 維持外觀和照護身體﹙如皮膚、臉部、牙齒、頭皮，指甲和生殖器﹚？ | 0 | 1 | 1 | 2 |
| 21 | 使用廁所（如去浴室、穿脫衣服/尿布或洗澡）？ | 0 | 1 | 1 | 2 |
| 22 | 在沒有協助的情況下從頭到腳脫衣服（例如挑衣服、扣鈕扣和拉拉鍊、綁腰帶）？ | 0 | 1 | 1 | 2 |
| 23 | 自主飲食（包括使用叉子、刀子、湯匙、開瓶蓋、罐頭、裝袋） | 0 | 1 | 1 | 2 |
| 24 | 自主照顧您的福祉和健康（如管理日常用藥、身體活動、預約門診） | 0 | 1 | 1 | 2 |

**參與量表(Participation scale)**

|  | 您有什麼問題/困難嗎？ | 沒有問題  (0-4%) | 輕/中度  (5-49%) | 重度  (50-95%) | 完全困難  (96-100%) |
| --- | --- | --- | --- | --- | --- |
| 1 | 在不同領域的日常生活中幫助需要幫助的他人？ | 0 | 1 | 1 | 1 |
| 2 | 以適當的社交禮儀內容和方式（如以擁抱作為問候）與他人身體接觸？ | 0 | 1 | 1 | 2 |
| 3 | 與他人交往和維繫社會關係？ | 0 | 1 | 1 | 2 |
| 4 | 按照您個人的期望參加社區生活？ | 0 | 1 | 1 | 2 |
| 5 | 參與遊戲（如撲克牌、記憶遊戲、棋盤遊戲）？ | 0 | 1 | 1 | 2 |
| 6 | 參與運動？ | 0 | 1 | 1 | 2 |
| 7 | 實踐您的文化興趣？ | 0 | 1 | 1 | 1 |
| 8 | 做手工藝或針線活？ | 0 | 1 | 1 | 1 |
| 9 | 追求您的愛好？ | 0 | 1 | 1 | 2 |
| 10 | 參加社交聚會/與親友相聚一起 | 0 | 1 | 1 | 2 |
| 11 | 參與您的宗教信仰活動？ | 0 | 1 | 1 | 2 |
