# Supplementary material for: Psychometric validation of the Chinese version of the PaArticular Scales among elderly residents in long-term care facilities with joint contractures
Source: BMC Geriatr. 2021 Jun 9;21:353. doi: 10.1186/s12877-021-02297-5 (PMC8190856; doi:10.1186/s12877-021-02297-5)
Supplement: Supplementary file 4 — Additional file 4: [file 12877_2021_2297_MOESM4_ESM.docx]

A detailed description of the Chinese version compared to the original version

Since the PaArticular Scales were introduced, two versions of the scale have been used—the ‘German version’ and the ‘English version’, which have slight differences in format and item numbers. With the development of the Chinese version, a slight format difference emerged between the English version and the Chinese version. Below, we explain the differences between the two versions, including the pros and cons of the Chinese version developed in the present study.

1. English version vs. Chinese Version - Design Comparison

The design frameworks of the English version and the Chinese version are the same and share the same ICF (International Classification of Functioning, Disability and Health) design as the original German version. Both design frameworks were developed according to the ICF. The only evident changes are the item numbers and the “Core Sets” with a category layout. Unlike the English version, the advantage of the Chinese version is that it has a matching relationship with the ICF Core Sets and is clearly displayed on the form.

2. English version vs. Chinese Version - Context Comparison

Both versions have the same scale range, number of items, and subscale features. The Chinese version has two more items and “Core Sets” than the English version. The two additional items are “Activities” and “Participation”, similar to the previous English version.

3. English version vs. Chinese Version - Development Comparison

The design frameworks of the English version and the Chinese version are the same. However, different strategies can be used for development; the Chinese version was developed from the English version through five steps: translation, review, back-translation, review by a panel of specialists, and a pretest. The global version is already in English. The English version was developed at a consensus conference held in southern Germany in May 2014. National experts developed an ICF and a health-based standard set consisting of 105 categories for the Activities and Participation components.
