# Supplementary material for: Psychometric validation of the Chinese version of the PaArticular Scales among elderly residents in long-term care facilities with joint contractures
Source: BMC Geriatr. 2021 Jun 9;21:353. doi: 10.1186/s12877-021-02297-5 (PMC8190856; doi:10.1186/s12877-021-02297-5)
Supplement: Supplementary file 5 — Additional file 5: [file 12877_2021_2297_MOESM5_ESM.docx]

*The World Health Organization Quality of Life (WHOQoL)-BREF*

這份問卷詢問您對於自己的生活品質、健康、以及其他生活領域的感覺。請您回答所有的問題。如果您對某一問題的回答不確定，請選出五個答案中最適合的一個，通常會是您最早想的那個答案。

我們的問題所關心的是您**最近兩星期內**的生活情形，請您用自己的標準、希望、愉快、以及關注點來回答問題。請參考下面的例題：

例題一：整體來說，您滿意自己的健康嗎？

□極不滿意 □不滿意 □中等程度滿意 □滿意 □極滿意

請選出最適合您在**最近兩星期內**對自己健康的滿意程度，如果您極滿意自己的健康，就在「極滿意」前的□內打「」。請詳細閱讀每個題目，並想想您自己的感覺，然後就每一個題目選出最適合您的答案。謝謝您的協助！

1.整體來說，您如何評價您的生活品質？

□極不好 □不好 □中等程度好 □好 □極好

2.整體來說，您滿意自己的健康嗎？

□極不滿意 □不滿意 □中等程度滿意 □滿意 □極滿意

3.您覺得身體疼痛會妨礙您處理需要做的事情嗎？

□完全沒有妨礙 □有一點妨礙 □中等程度妨礙 □很妨礙 □極妨礙

4.您需要靠醫療的幫助應付日常生活嗎？

□完全沒有需要 □有一點需要 □中等程度需要 □很需要 □極需要

5.您享受生活嗎？

□完全沒有享受 □有一點享受 □中等程度享受 □很享受 □極享受

6.您覺得自己的生命有意義嗎？

□完全沒有 □有一點有 □中等程度有 □很有 □極有

7.您集中精神的能力有多好？

□完全不好 □有一點好 □中等程度好 □很好 □極好

8.在日常生活中，您感到安全嗎？

□完全不安全 □有一點安全 □中等程度安全 □很安全 □極安全

9.您所處的環境健康嗎？(如污染、噪音、氣候、景觀)

□完全不健康 □有一點健康 □中等程度健康 □很健康 □極健康

10.您每天的生活有足夠的精力嗎？

□完全不足夠 □少許足夠 □中等程度足夠 □很足夠 □完全足夠

11.您能接受自己的外表嗎？

□完全不能夠 □少許能夠 □中等程度能夠 □很能夠 □完全能夠

12.您有足夠的金錢應付所需嗎？

□完全不足夠 □少許足夠 □中等程度足夠 □很足夠 □完全足夠

13.您能方便得到每日生活所需的資訊嗎？

□完全不方便 □少許方便 □中等程度方便 □很方便 □完全方便

14.您有機會從事休閒活動嗎？

□完全沒有機會 □少許機會 □中等程度機會 □很有機會 □完全有機會

15.您四處行動的能力好嗎？

□完全不好 □有一點好 □中等程度好 □很好 □極好

16.您滿意自己的睡眠狀況嗎？

□極不滿意 □不滿意 □中等程度滿意 □滿意 □極滿意

17.您對自己從事日常活動的能力滿意嗎？

□極不滿意 □不滿意 □中等程度滿意 □滿意 □極滿意

18.您滿意自己的工作能力嗎？

□極不滿意 □不滿意 □中等程度滿意 □滿意 □極滿意

19.您對自己滿意嗎？

□極不滿意 □不滿意 □中等程度滿意 □滿意 □極滿意

20.您滿意自己的人際關係嗎？

□極不滿意 □不滿意 □中等程度滿意 □滿意 □極滿意

21.您滿意自己的性生活嗎？

□極不滿意 □不滿意 □中等程度滿意 □滿意 □極滿意

22.您滿意朋友給您的支持嗎？

□極不滿意 □不滿意 □中等程度滿意 □滿意 □極滿意

23.您滿意自己住所的狀況嗎？

□極不滿意 □不滿意 □中等程度滿意 □滿意 □極滿意

24.您對醫療保健服務的方便程度滿意嗎？

□極不滿意 □不滿意 □中等程度滿意 □滿意 □極滿意

25.您滿意所使用的交通運輸方式嗎？

□極不滿意 □不滿意 □中等程度滿意 □滿意 □極滿意

26.您常有負面的感受嗎？（如傷心、緊張、焦慮、憂鬱等）

□從來沒有 □不常有 □一半有一半沒有 □很常有
